# Supplementary material for: Abundance and morphology of charcoal in sediments provide no evidence of massive slash-and-burn agriculture during the Neolithic Kuahuqiao culture, China
Source: PLoS One. 2020 Aug 19;15(8):e0237592. doi: 10.1371/journal.pone.0237592 (PMC7437897; doi:10.1371/journal.pone.0237592)
Supplement: Attachment — Submitted filename: Response to Reviewers.docx [file pone.0237592.s002.docx]

Dear Dr. Biehl,

We appreciate the time that you took in addressing our manuscript. In our revision we have addressed all the main concerns of the reviewers and have made the requested specific changes based on the reviewers’ comments. Specifically, we have performed more experiments to collect additional data on plant taxa and time period of interest. Furthermore, we have made extensive edits to improve the readability of the manuscript. You will see our responses to the comments below. We appreciate these suggestions and think they significantly improve the manuscript and hope that you find that our manuscript is now appropriate for publication.

We thank you for your time and help in making this paper much clearer to readers and look forward to hearing from you. We are happy to make additional revisions if necessary.

Sincerely,

Bin Zhou

Review Comments to the Author

Please use the space provided to explain your answers to the questions above. You may also include additional comments for the author, including concerns about dual publication, research ethics, or publication ethics. (Please upload your review as an attachment if it exceeds 20,000 characters)

Reviewer #1: Zhou et al.’s paper presents an interesting macrocharcoal-based evidence to argue the previous hypothesis that whether the slash-and-burn practice was used in early rice farming in the low-lying coastal Kuahuqiao area 8-7ka. The simulation experiment using common modern plants for burning successfully established the useful criteria to morphologically discriminate charcoal between herbs and woods. Their result shows that there was no regional human-induced vegetation burning for rice agriculture at Kuahuqiao, which agrees well with pollen analysis at the archaeological site. This study offers a significant insight regarding the early rice farming in coastal plains.

Reply: We thank the reviewer for taking the time to evaluate our manuscript and providing constructive comments, which contributed to the improvement of the manuscript.

Several questions should be addressed before publication.
1) The incomplete selected plant list for the burning experiment
The study site is located in the monsoonal climate under which zonal broadleaved evergreen forests flourish. Based pollen results (Zong et al., 2007; Innes et al., 2009; Shu et al., 2010, 2012) show that Pinus and Cyclobalanopsis, important trees, contribute a large proportion into pollen flora. However, these two taxa are ignored in the authors’ combustion experiment. Please tell the reason why these important trees are lack.

Reply: We thank the reviewer for this suggestion. We have performed additional combustion experiments and included *Pine, Cyclobalanopsis, Oryza* stem.

2) Low sampling resolution
Zhou et al. utilized only 13 sediment samples for macro-charcoal analysis collected from the combined 34m-long cores. Even worse is that merely two samples from 8240-7450 cal.aBP corresponding to the cultural layers at the archaeological site. The low resolution definitely weakens the conclusion they drew to argue against the slash-and-burn hypothesis. I strongly suggest more samples are highly necessary esp. during the 8-7ka interval.

Reply: We have added three additional data points during the 8-7 ka, and the result is now shown in the revised manuscript. These new results did not change our conclusion.

3) Abundance herbaceous charcoal around 8550 cal. a BP.
The sedimentary charcoal shows high concentration of herbaceous charcoal near the 8550 years ago. The authors provide a reasonable explanation for the exceptional high content that the tide environment encouraged herbs to colonize the tidal flat. However, is it a common situation in coastal area? Please provide published examples to support this idea in order to convince the readers.

Reply: We thank the reviewer for this suggestion and have now added references to support this idea. Please check lines of 367-369, and reference 37.

Minor mistakes or questions:
1) lines 95-101, “northern boundary of the subtropical climate zone” could be central subtropical zone; “dominated by north subtropical evergreen deciduous ” north corrected to central; Pinus Linn, Cyclobalanopsis oerst? Correct to Pinus, Cyclobalanopsis. Gramineae to Gramineae or Line

Reply: We have made the correction in lines 95-101.

2) Line 114, Brassica chinensis is an important economical oil-producing plant and was introduced into China during late historical time. Thus, it is impropriate to burn it for charcoal analysis.

Reply: We meant “Weeds from tidal flats”. We apologize for this error and have made the modification in lines 114.

3) Line 117, “a mixture of Wood (simplified as Wood).” Wood here is confusing. What are included? Please detail it.

Reply: We have added the details— “the trunk of arbor including *Cinnamomum* and *Quercus”*. Modification has been made in the figures and article.

4) Line 141, “seven plant residues of KHQ-1502 and two plant residues of KHQ-14 were” nine samples was dated. However, 8 ages are indicated in Fig.6, why

Reply: We apologize for the omission. We have added the age data to Fig.6.

5）Fig.3, only 13 taxa plant are shown. However, 18 taxa are selectedd for combustion experiment. Why? Moreover, Artemisia in southern China are mainly in herb form instead of trees. Also, Bamboos are usually assigned into poaceaous woods.

Reply: We have added three additional taxa for the combustion and fragmentation experiments. We performed fragmentation experiments on the majority (16 out of 21) but not all of selected taxa because fragmentation showed relatively consistent effects on all samples. We included the major and representative taxa of the study area, which should be sufficient to address the research objective.

Following this suggestion, we assigned *Phyllostachys* as the trunk of woods. Because the Artemisia we collected in the field were tall and their stems have become lignified, we classified it as a shrub according to botanical definition.

Reviewer #2: As a direct product of fire and vegetation, paleocharcoal is one of the most important evidence to reconstructing the fire history and ancient human activities. However, it is still controversial whether morphological features of charcoal can be used as indicators to identifying plant species. In this manuscript, the authors innovatively conducted combustion and fragmentation conditional experiments based on modern representative reference to simulate fire and depositional processes. They established morphological criteria of charcoal from modern vegetation to distinguish herbaceous and woody plants, and further applied them to sediment cores adjacent to the archaeological site revealing local environment changes and early human activities, providing new insight about slash-and-burn farming activities in lower Yangtze region in the early Holocene. This case study provides more evidence to further illustrating the relationship between the production, deposition of charcoal and farming activity in the early stage of agriculture.

The word in line 29 "then applied to sediment cores (KQH-14/15)", "KQH" should be "KHQ"?

Reply: We thank the reviewer for taking the time to evaluate our manuscript and providing constructive comments, which have helped to improve the manuscript. We have made the correction in line 29.
